# Soluble CD163 and incident cardiovascular events in patients with systemic lupus erythematosus: An observational cohort study

**DOI:** 10.1111/joim.13490

**Published:** 2022-04-10

**Authors:** Clémence David, Nathalie Costedoat‐Chalumeau, Drifa Belhadi, Cedric Laouénan, Anne Boutten, Julie Chezel, Diane Rouzaud, Monique Dehoux, Véronique Le Guern, Alexis Mathian, Sébastien de Almeida Chaves, Pierre Duhaut, Olivier Fain, Lionel Galicier, Pascale Ghillani‐Dalbin, Jean Emmanuel Kahn, Nathalie Morel, Laurent Perard, Micheline Pha, Francoise Sarrot‐Reynauld, Olivier Aumaitre, François Chasset, Nicolas Limal, Helene Desmurs‐Clavel, Felix Ackermann, Zahir Amoura, Thomas Papo, Karim Sacre

**Affiliations:** ^1^ Département de Médecine Interne Hôpital Bichat Assistance Publique Hôpitaux de Paris (APHP) Institut national de la santé et de la recherche médicale (INSERM) U1149 Université de Paris Paris France; ^2^ APHP, Hôpital Cochin, Département de Médecine Interne Centre de Reference Maladies Auto‐immunes et Systémiques Rares Université de Paris, CRESS, INSERM, INRA Paris France; ^3^ Departement d'Epidémiologie et de Recherche Clinique Hôpital Bichat APHP Université de Paris Paris France; ^4^ Département de Biochimie Hôpital Bichat, APHP Université de Paris Paris France; ^5^ Sorbonne Université, Assistance Publique–Hôpitaux de Paris Groupement Hospitalier Pitié–Salpêtrière French National Referral Center for Systemic Lupus Erythematosus Antiphospholipid Antibody Syndrome and Other Autoimmune Disorders Service de Médecine Interne 2 Paris France; ^6^ Département de Médecine Interne, Hôpital Purpan Centre Hospitalo‐Universitaire (CHU) de Toulouse Toulouse France; ^7^ Département de Médecine Interne Hôpital Amiens Nord CHU d'Amiens Amiens France; ^8^ Département de Médecine Interne Hôpital Saint Antoine APHP Université Pierre et Marie Curie Paris France; ^9^ Département d'Immunologie Clinique Hôpital Saint Louis APHP Université de Paris Département de Médecine Interne Hôpital Saint Joseph Marseille France; ^10^ Département de Immunologie Hôpital Pitié‐Salpétrière, APHP Université Pierre et Marie Curie Paris France; ^11^ Département de Médecine Interne Hôpital Ambroise Paré APHP Université de Versailles‐Saint‐Quentin en Yvelines France; ^12^ Département de Médecine Interne Hôpital St Joseph St Luc Lyon France; ^13^ Département de médecine interne Hôpital Michallon CHU de Grenoble Alpes Grenoble France; ^14^ Département de médecine interne Hôpital Gabriel‐Montpied CHU de Clermont‐Ferrand France; ^15^ Département de médecine interne Hôpital Tenon APHP Université Pierre et Marie Curie Paris France; ^16^ Département de médecine interne Hôpital Henri Mondor APHP Université Paris‐Est Créteil Paris France; ^17^ Département de médecine interne Hôpital Edouard Herriot Hospices Civils de Lyon Lyon France; ^18^ Département de médecine interne Hôpital Foch Suresnes France

**Keywords:** systemic lupus erythematosus, cardiovascular diseases, biomarkers, CD163

Dear Editor,

The increased prevalence of cardiovascular events (CVE) in systemic lupus erythematosus (SLE) is not fully explained by traditional risk factors. Accordingly, prediction models such as Framingham score are not accurate at identifying CVE risks in this population [1]. sCD163, the soluble form of the scavenger receptor CD163 on macrophage, have been implicated in several inflammatory or autoimmune diseases including SLE [2]. Our group previously showed that sCD163 is a biomarker of accelerated atherosclerosis in SLE patients [3]. The aim of this study was to determine whether sCD163 was associated with incident CVE in SLE.

All SLE patients included in the randomized, double‐blind, placebo‐controlled, multicenter PLUS trial were screened [4]. Patients without history of CVE at inclusion and with a follow‐up period of >20 months were analyzed [5]. sCD163 level was measured using enzyme‐linked immunosorbent assay on serum collected at PLUS inclusion. The primary outcome was the incident CVE (see Supporting Information).

Among the 573 SLE patients of the PLUS study, 442 (37 [IQR: 29–48]; 90.5% female) were analyzed for the primary outcome with a median follow up of 110 (IQR: 99–120) months (Fig. S1). Ninety‐nine (22.4%) were smokers, 60 (13.6%) had hypertension, 51 (11.6%) a BMI >30 kg/m^2^, 34 (7.7%) dyslipidaemia and 11 (2.5%) diabetes. No patients had an eGFR <60 mL/min/1.73 m^2^. Antiphospholipid antibodies were found in 217 (49.1%) patients. The median duration of SLE disease at baseline was of 7 (IQR: 3–12) years (Table S1).

Overall, 29 (6.6%) patients experienced at least one CVE that occurred at a median of 67 (IQR: 31–91) months after inclusion. CVE included coronary heart disease (*n* = 14), ischemic stroke (*n* = 11), peripheral arterial disease requiring revascularization (*n* = 2), aortic aneurysm requiring surgery (*n* = 1) and sudden cardiac death (*n* = 1). Six patients had more than one CVE.

At PLUS inclusion, the median level of sCD163 in serum was 324.1 ng/ml (IQR: 218.4–470.1) (Fig. S2). By using maximally selected Log‐Rank statistic, the cut point in sCD163 value that provided the best separation between the SLE patients who developed CVE and those who did not was 263 ng/ml (Fig. S3). In a multivariate Cox regression model with CVE as the dependent variable, dyslipidemia (HR 3.0 [95% CI: 1.2–7.5]), age (HR 1.7 [95% CI: 1.3–2.3]), and sCD163> 263 ng/ml (HR 2.7 [95% CI: 1.1–7.0]) were associated with the occurrence of CVE (Table S2). Accordingly, Kaplan–Meier analysis showed that a concentration of sCD163 >263 ng/ml in serum at inclusion was associated with the occurrence of CVE during follow up (Fig. 1). Interestingly, multivariate Cox regression model with sCD163 >263 ng/ml as the dependent variable showed that BMI (OR 1.1 [95% CI: 1.0–1.1]), SLEDAI (OR 1.1 [95% CI: 1.0‐1.3]) and the use of immunosuppressive drugs (OR 1.6 [95% CI: 1.1–2.4]) were associated with increased sCD163 (Table S3). In addition, sCD163 appeared to correlate with the SLEDAI activity score at inclusion (Fig. S4).

**Fig. 1:**
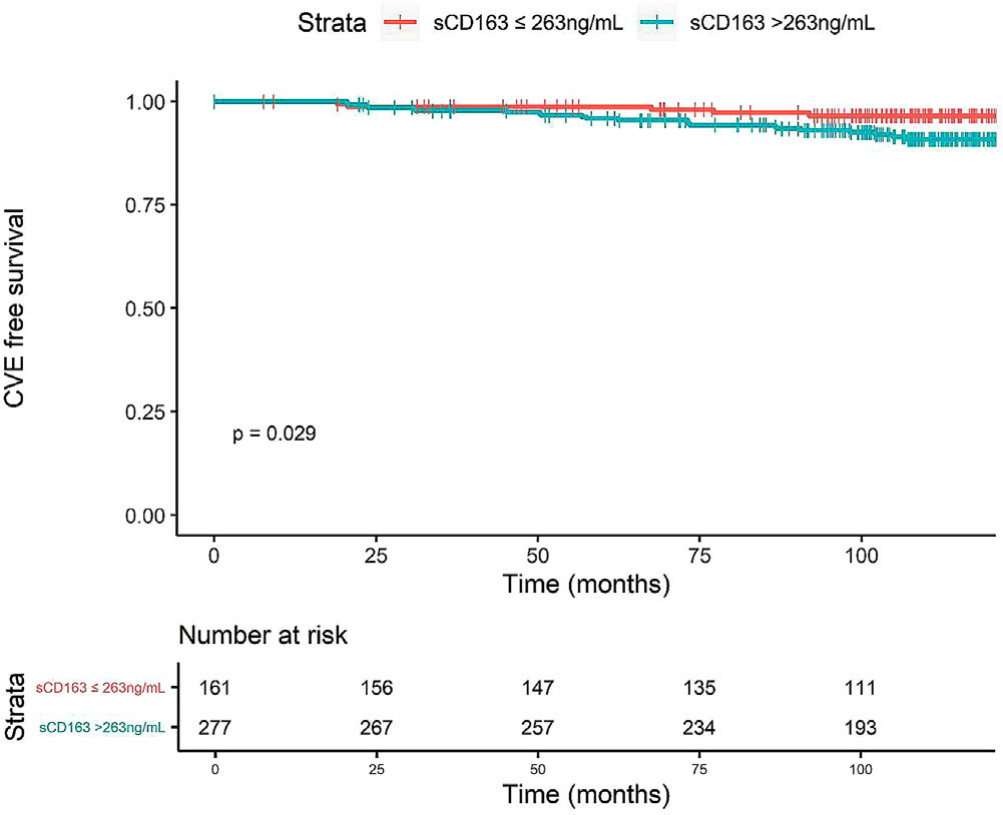
Kaplan–Meier curves reflecting cumulative proportion of systemic lupus erythematosus (SLE) patients free of CVE according to sCD163 level. Analysis was performed on 438 patients. Abbreviation: CVE, cardiovascular event.

The present study demonstrates that a high level of sCD163—with a cut‐off value of 263 ng/ml—is associated with incident CVE in SLE patients.

sCD163 may reflect SLE activity as suggested by its association with SLEDAI score and the use of immunosuppressive drugs. In the general population, CD163^+^ macrophages contribute to atherosclerotic lesions and plaque progression [6]. In SLE, CD163^+^ macrophages have been implicated in the pathogenesis of lupus nephritis [2]. High sCD163 level in serum may thus reflect the involvement of CD163^+^ macrophages in both atherosclerosis and SLE and indicate the implication of SLE immune dysregulation in atherosclerotic inflammatory process [7, 8]. In our cohort, sCD163—known to be coupled with impaired insulin sensitivity and obesity [9]—was associated with BMI. Accordingly, sCD163 may reflect the chronic low‐grade inflammation arising from adipose tissue macrophage that contribute—overweight being a strong contributor to lupus‐associated atherosclerosis [10]—to the burden of CVE in SLE.

Our study has some limits: the primary outcome was not available for all PLUS patients, the small number of CVE limits statistical power and sCD163 was only assessed once at inclusion.

Moreover, the cut‐off value of 263 ng/ml showed a good sensitivity (86.2%) but a poor specificity (37.5%) for CVE in SLE. Accordingly, such cut‐off value seems more efficient to identify SLE patients at very low risk of CVE. Our study has also several strengths. It is an ancillary longitudinal study of a clinical trial including more than 400 SLE with an extended follow‐up. Its primary endpoint focuses on incident CVE in contrast to most studies assessing the risk for CVE in SLE.

Macrophage‐specific sCD163 serum level reflects lupus disease activity and is associated with CVE in SLE patients at apparent low cardiovascular risk. Stratifying patients according to sCD163 levels may help tailoring preventive treatment of SLE‐related atheroma with statin or aspirin.

## Conflict of interest

The authors declare that they have no conflict of interest.

## Author contributions

Conceptualization; data curation; formal analysis; funding acquisition; investigation; methodology; project administration; resources; supervision; validation; writing–original draft; and writing–review and editing: Nathalie Costedoat‐Chalumeau and Karim Sacre. Formal analysis; investigation; methodology; software; validation; writing–original draft; and writing–review and editing: Drifa Belhadi. Formal analysis; investigation; methodology; resources; validation; writing–original draft; and writing–review and editing: Anne Boutten. Formal analysis; investigation; methodology; writing–original draft; writing–review and editing: Julie Chezel and Diane Rouzaud. Conceptualization; investigation; methodology; writing–original draft; and writing–review and editing: Monique Dehoux. Resources and writing–review and editing: Alexis Mathian. Investigation; resources; and writing–review and editing: Sébastien De Almeida Chaves, Pierre Duhaut, Olivier Fain, Pascale Ghillani‐Dalbin, Nathalie Morel, Laurent Perard, Micheline Pha, Nicolas Lima, and Felix Ackermann. Investigation; resources; writing–original draft; and writing–review and editing: Lionel Galicier. Resources and writing–review and editing: Zahir Amoura. Resources; writing–original draft; and writing–review and editing: Thomas Papo.

## Funding information

French PHRC 2005 (number 05–125) Assistance Publique Hôpitaux de Paris; French CRC 2017 (number 17–068) Assistance Publique Hôpitaux de Paris; Assistance Publique Hôpitaux de Paris (Année‐Recherche 2019); Fondation pour la Recherche Medicale (FRM DEA20170638077).

## Ethics statement

The study was approved by the Comité de Protection des Personnes, St Louis Hospital, Paris (PLUS) and by the Comité de Protection des Personnes SUD‐EST II, Lyon. All participants gave written informed consent to participate at the time of study enrollment.

## Supporting information


**Figure S1**: Flow chart.
**Figure S2**: sCD163 concentration in SLE patients.
**Figure S3**: Maximally selected Log‐Rank statistic for the cutpoint in sCD163 value.
**Figure S4**: sCD163 in SLE patients correlates with SLEDAI.
**Table S1**: Characteristics of SLE patients at inclusion.
**Table S2**: Risk factors for cardiovascular events in SLE patients.
**Table S3**: Risk factors for high sCD163 in SLE patients.
