# Supplementary material for: Soluble CD163 and incident cardiovascular events in patients with systemic lupus erythematosus: An observational cohort study
Source: J Intern Med. 2022 Apr 10;292(3):536–9. doi: 10.1111/joim.13490 (PMC9542031; doi:10.1111/joim.13490)
Supplement: Supplementary file 1 — Figure S1: Flow chart. Figure S2: sCD163 concentration in SLE patients. Figure S3: Maximally selected Log‐Rank statistic for the cutpoint in sCD163 value. Figure S4: sCD163 in SLE patients correlates with SLEDAI. Table S1: Characteristics of SLE patients at inclusion. Table S2: Risk factors for cardiovascular events in SLE patients. Table S3: Risk factors for high sCD163 in SLE patients. [file JOIM-292-536-s001.docx]

**SUPPLEMENTARY MATERIAL**

**List of collaborators - PLUS study group:**

Leonardo Astudillo, Cristina Belizna, Nadia Belmatoug, Olivier Benveniste, Audrey Benyamine, Holly Bezanahary, Patrick Blanco, Benoît Brihaye, Patrice Cacoub, Emmanuel Chatelus, Judith Cohen-Bittan Richard Damade, Eric Daugas, Christian de-Gennes, Jean-François Delfraissy, Céline Delluc, Aurélien Delluc, Alain Dupuy, Isabelle Durieu, Hang-Korng EA, Dominique Farge, Camille Frances, Christian Funck-Brentano, Frédérique Gandjbakhch, Justine Gellen-Dautremer, Bertrand Godeau, Cécile Goujard, Catherine Grandpeix, Claire Grange, Gaëlle Guettrot-Imbert, Loïc Guillevin, Eric Hachulla, Jean-Robert Harle, Julien Haroche, Pierre Hausfater, Jean Sebastien Hulot, Moez Jallouli, Jean Jouquan, Gilles Kaplanski, Homa Keshtmand, Mehdi Khellaf, Olivier Lambotte, David Launay, Du Le Thi Huong, Gaelle Leroux, Hervé Levesque, Olivier Lidove, Frederic Liote, Eric Liozon, Kim Ly, Matthieu Mahevas, Kubéraka Mariampillai, Xavier Mariette, Karin Mazodier, Marc Michel, Luc Mouthon, Rokiya Ngack, Jacques Ninet, Eric Oksenhendler, Jean-Luc Pellegrin, Olivier Peyr, Anne-Marie Piette, Jean-Charles Piette,Vincent Poindron, Fabienne Roux, David Saadoun, Sabrinel Sahali, Laurent Sailler, Bernadette Saint-Marcoux, Yoland Schoindre, Jérémie Sellam, Damien Sene, Jacques Serratrice, Pascal Seve, Jean Sibilia, Claude Simon, Amar Smail, Christelle Sordet, Jérôme Stirnemann, Benjamin Terrier, Salim Trad, Jean-François Viallard, Pierre-Jean Weiller, Noël Zahr.

**PATIENTS AND METHODS**

**Patients**

This ancillary study is derived from the previously published PLUS study [1] which involved 573 systemic lupus erythematosus (SLE) patients in 37 centres from June 2007 through August 2010 in France. Details of the PLUS study methods including inclusion and exclusion criteria, data collection and definitions of associated condition have been previously published [1]. SLE patients with extended follow-up period (> 20 months) and no past history of cardiovascular event (CVE) at PLUS inclusion were analyzed (**Figure S1**) [2]. SLE subjects were considered to have hypertension if they repeatedly had a systolic blood pressure of at least 140 mm Hg or a diastolic blood pressure of at least 90 mm Hg. Patients were considered to have dyslipidemia if they had LDL-cholesterol >116 mg/dL [3]. The diagnosis of antiphospholipid syndrome was based on a history of venous and/or arterial thromboses or recurrent miscarriages in the presence of antiphospholipid antibodies in accordance with published criteria [4]. Lupus nephritis diagnosis was based on International Society of Nephrology/Renal Pathology Society classification [5]. The study was approved by the Comité de Protection des Personnes, St Louis Hospital, Paris (PLUS) and by the Comité de Protection des Personnes SUD-EST II, Lyon. All participants gave written informed consent to participate at the time of study enrollment.

**Soluble CD163 measurement**

All measurements were performed on serum samples collected at PLUS inclusion, aliquoted and stored at -80°C and thawed just before testing in August 2018. Serum sCD163 was measured on a 10-fold dilution by a specific sandwich enzyme-linked immunosorbent assay according to the manufacturer’s instructions (DY1607 ELISA DuoSet® Quantikine® R&D Systems Europe, Abingdon, UK).

**Primary outcome**

The primary outcome was the occurrence of CVE during follow-up. CVE was defined as coronary heart disease, (i.e. hospitalization for unstable angina, coronary revascularization, or myocardial infarction), stroke, revascularization procedure for other atherosclerotic cardiovascular diseases or sudden cardiac death. CVE were ascertained through analysis of medical records and using a standardized questionnaire by a physician blinded to sCD163 results. All CVE that occurred through March 2019 were considered for analysis. For patients who had more than 1 incident CVE, only the first event was considered for analysis.

**Statistical analysis**

Continuous variables were expressed as median [interquartile range (IQR)] and categorical variables were expressed as number and percentage. Wilcoxon test was used to compare the median level of sCD163 and the occurrence of CVE. The ‘Maximally Selected Log-Rank statistic’ method [6] was used to calculate the optimal threshold of sCD163 level to predict a major CVE. Univariate Cox models were used to determine the association (hazard ratios with 95% confidence intervals) between clinical, biological and therapeutic factors and CVE (primary outcome) and sCD163. Factors identified in univariate analysis with a p-value <0.2 were selected to build a multivariate Cox regression model using a backward selection procedure to identify those independently associated with CVE (primary outcome) and sCD163. The risk of CVE stratified by the calculated sCD163 level threshold was analysed using the Kaplan–Meier method and groups were compared using a log-rank test. The Kendall rank correlation test was used to determine correlations between variables, with R being the Kendall correlation coefficient.

**TABLES**

**Table S1 Characteristics of SLE patients at inclusion**

|  | Missing data, n (%) | SLE Patients, n=442 |
| --- | --- | --- |
| Age, years | 0 (0) | 37 (29;48) |
| Female gender, n (%) | 0 (0) | 400 (90.5) |
| Current smoker, n (%) | 1 (0.2) | 99 (22.4) |
| Diabetes, n (%) | 0 (0) | 11 (2.5) |
| LDL-cholesterol, mg/dl | 64 (14.5) | 100 (80;120) |
| Dyslipidaemia, n (%) | 0 (0) | 34 (7.7) |
| Hypertension, n (%) | 0 (0) | 60 (13.6) |
| BMI, kg/m2 | 1 (0.2) | 23 (21;26) |
| BMI>30 kg/m², n (%) | 1 (0.2) | 51 (11.6) |
| eGFR<60 ml/mn/1,73m2, n (%)* | 13 (2.9) | 0 (0) |
| Duration of SLE disease, years | 0 (0) | 7 (3-12) |
| Lupus nephritis, n (%) | 0 (0) | 36 (8.1) |
| aPL antibodies, n (%) | 0 (0) | 217 (49.1) |
| APS, n (%) | 0 (0) | 71 (16.1) |
| SLEDAI score | 0 (0) | 2 (0-2) |
| SLEDAI>4 | 0 (0) | 47 (10.6) |
| Steroids | 0 (0) | 384 (86.9) |
| Steroids daily dose at inclusion | 0 (0) | 8 (5;10) |
| Hydroxychloroquine, n (%) | 0 (0) | 442 (100) |
| Immunosuppressive drugs, n (%) | 0 (0) | 224 (50.7) |
| Biologics, n (%) | 0 (0) | 37 (8.4) |
| Antiplatelet treatment, n (%) | 0 (0) | 124 (28.1) |
| Statin, n (%) | 0 (0) | 29 (6.6) |
| ACE inhibitors, n (%) | 0 (0) | 81 (18.3) |
| Anticoagulant treatment, n (%) | 0 (0) | 76 (17.2) |
| Hormonal contraception, n (%) | 0 (0) | 146 (33) |

Results are shown as median (IQR) or n (%).

eGFR was calculated with the modification of diet in renal disease equation

Immunosuppressive drugs included azathioprine, methotrexate, ciclosporin, mycophenolate mofetil, cyclophosphamide, thalidomide, tacrolimus, lenalidomide or sulfasalazine.

Biologics included rituximab or belimumab.

* According to the inclusion criteria in the PLUS study

ACE: angiotensin converting enzyme; aPL: antiphospholipid; APS: antiphospholipid syndrome; BMI: body mass index; eGFR: estimated glomerular filtration rate; IQR: interquartile range; LDL: low-density lipoprotein, SLEDAI: Systemic Lupus Erythematosus Disease Activity Index

**Table S2 Risk factors for cardiovascular events in SLE patients**

|  | missing data | Univariate analysis | | Multivariate analysis | |
| --- | --- | --- | --- | --- | --- |
|  |  | HR (95% CI) | p | HR (95% CI) | p |
| Age (for 10-year aged) | 0 (0) | 1.8 (1.4 - 2.3) | <0.0001 | 1.7 (1.3 - 2.3) | 0.0002 |
| Female gender | 0 (0) | 0.8 (0.2 - 2.7) | 0.7336 | - |  |
| Current smoker | 1 (0.2) | 2.0 (0.9 - 4.2) | 0.0859 | - |  |
| Diabetes | 0 (0) | 1.2 (0.2 - 8.8) | 0.8588 | - |  |
| Dyslipidaemia | 0 (0) | 4.9 (2.4 - 9.8) | <0.0001 | 3.0 (1.2 - 7.5) | 0.0162 |
| Hypertension | 0 (0) | 2.9 (1.4 - 6.0) | 0.0045 | - |  |
| BMI, kg/m2 | 1 (0.2) | 1.0 (0.9 - 1.1) | 0.1277 | - |  |
| BMI>30, kg/m2 | 1 (0.2) | 1.7 (0.6 - 4.5) | 0.2745 | - |  |
| Duration of SLE disease | 0 (0) | 1.1 (1.0 - 1.1) | 0.0024 | - |  |
| Lupus nephritis | 0 (0) | 0.7 (0.2 - 2.9) | 0.5983 | - |  |
| aPL antibodies | 0 (0) | 1.5 (0.7 - 3.1) | 0.2985 | - |  |
| APS | 0 (0) | 1.9 (0.8 - 4.4) | 0.1159 | - |  |
| SLEDAI | 0 (0) | 1.0 (0.9 - 1.2) | 0.8758 | - |  |
| SLEDAI >4 | 0 (0) | 1.3 (0.4 - 3.8) | 0.6155 | - |  |
| Steroids | 0 (0) | 1.9 (0.5 - 8.2) | 0.3614 | - |  |
| HCQ ≥750ng/ml | 0 (0) | 0.7 (0.3 - 1.5) | 0.3419 | - |  |
| Immunosuppressive drug | 0 (0) | 1.6 (0.7 - 3.5) | 0.2153 | - |  |
| Biologics | 0 (0) | 1.7 (0.6 - 4.9) | 0.3274 | - |  |
| Hormonal contraception | 0 (0) | 0 (0 - Inf) | 0.9976 | - |  |
| sCD163 > 263 ng/mL | 4 (0.9) | 2.8 (1.1-7.3) | 0.0361 | 2.7 (1.0 - 7.0) | 0.0487 |

Analysis was performed on 442 patients

Immunosuppressive drugs included azathioprine, methotrexate, ciclosporin, mycophenolate, cyclophosphamide, thalidomide, tacrolimus, lenalidomide or sulfasalazine.

Biologics included rituximab or belimumab.

aPL: antiphospholipid; APS: anti phospholipid syndrome; BMI: body mass index; CI: confidence interval; HCQ, hydroxychloroquine level in blood; HR: hazard ratio; SLEDAI: Systemic Lupus Erythematosus Disease Activity Index

**Table S3 Risk factors for high sCD163 in SLE patients**

|  | missing data | Univariate analysis | | Multivariate analysis | |
| --- | --- | --- | --- | --- | --- |
|  |  | OR (95% CI) | p | OR (95% CI) | p |
| Age (for 10-year aged) | 0 (0) | 1.0 (0.9 - 1.2) | 0.9488 | - |  |
| Female gender | 0 (0) | 1.1 (0.5 - 2.0) | 0.8635 | - |  |
| Current smoker | 1 (0.2) | 0.9 (0.6 - 1.5) | 0.7444 | - |  |
| Diabetes | 0 (0) | 6.9 (1.4 - 124.3) | 0.0622 | - |  |
| Dyslipidaemia | 0 (0) | 1.5 (0.8 - 3.1) | 0.2368 | - |  |
| Hypertension | 0 (0) | 1.2 (0.8 - 1.9) | 0.4362 | - |  |
| BMI, kg/m2 | 1 (0.2) | 1.1 (1.0 - 1.1) | 0.0050 | 1.1 (1.0 - 1.1) | 0.0179 |
| Duration of SLE disease | 0 (0) | 1.0 (0.9 - 1.0) | 0.4076 | - |  |
| Lupus nephritis | 0 (0) | 2.2 (1.0 - 5.2) | 0.0625 | - |  |
| aPL antibodies | 0 (0) | 1.1 (0.8 - 1.6) | 0.5860 | - |  |
| APS | 0 (0) | 1.2 (0.7 - 2.0) | 0.5580 | - |  |
| SLEDAI | 0 (0) | 1.2 (1.1 - 1.3) | 0.0015 | 1.1 (1.0 - 1.3) | 0.0069 |
| Steroids | 0 (0) | 1.0 (0.6 - 1.8) | 0.9441 | - |  |
| HCQ | 0 (0) | 1.0 (1.0 – 1.0) | 0.0715 | - |  |
| Immunosuppressive drugs | 0 (0) | 1.8 (1.2 - 2.7) | 0.0022 | 1.6 (1.1 - 2.4) | 0.0214 |
| Biologics | 0 (0) | 2.3 (1.1 - 5.6) | 0.0391 | - |  |
| Hormonal contraception | 0 (0) | 1.1 (0.7 - 1.6) | 0.7146 | - |  |

Analysis was performed on 438 patients

Immunosuppressive drugs included azathioprine, methotrexate, ciclosporin, mycophenolate, cyclophosphamide, thalidomide, tacrolimus, lenalidomide or sulfasalazine.

Biologics included rituximab or belimumab.

aPL: antiphospholipid; APS: anti phospholipid syndrome; BMI: body mass index; CI: confidence interval; HCQ, hydroxychloroquine level in blood; OR: odds ratio; SLEDAI: Systemic Lupus Erythematosus Disease Activity Index

**FIGURES**

**Figure S1 Flow chart**


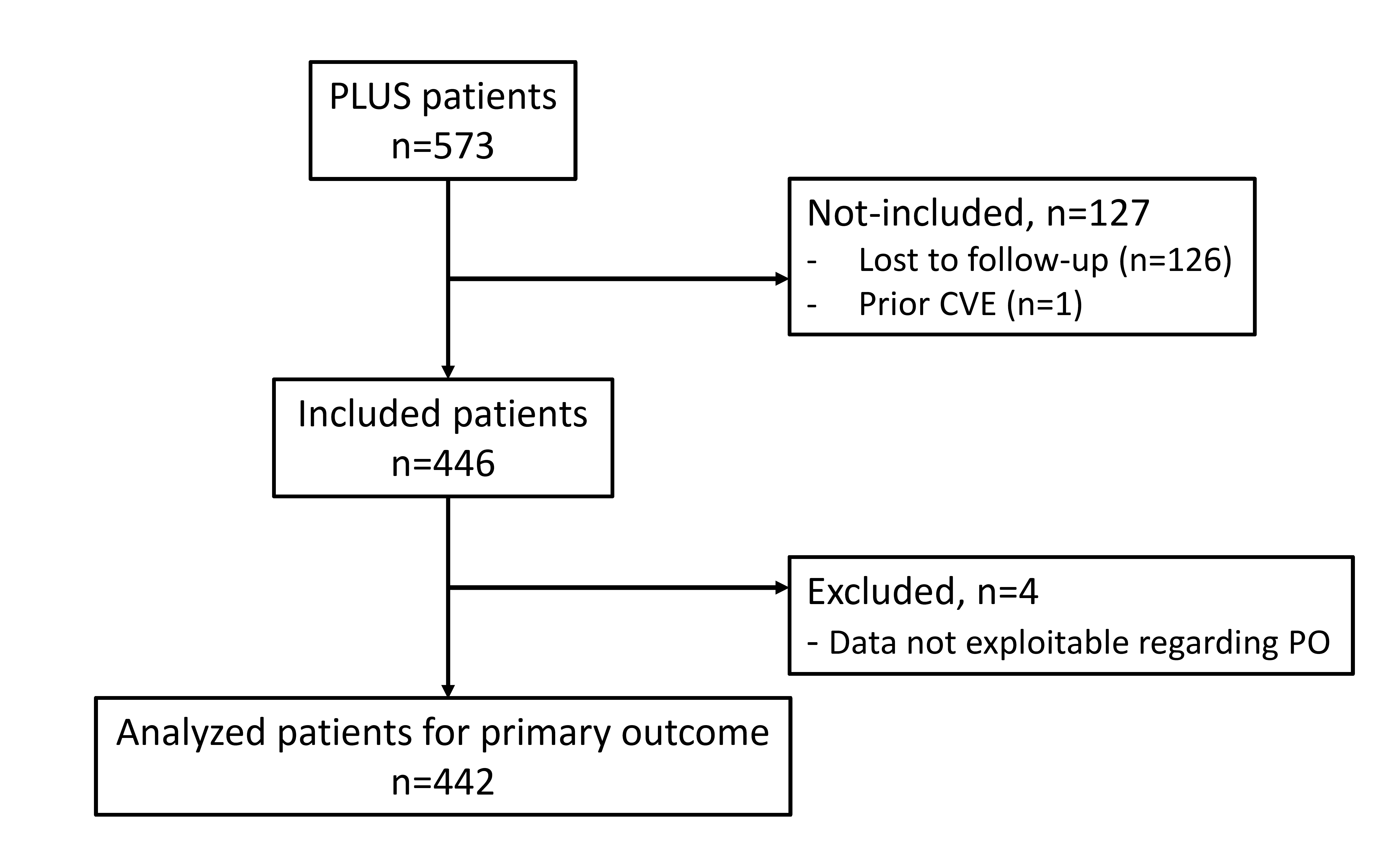


A total of 573 patients were included in the PLUS study from June 2007 through August 2010 at 37 centres in France [1]. Among them. 442 were eventually included in the study and analyzed for the primary outcome [2].

CVE. cardiovascular event

PO. primary outcome

**Figure S2 sCD163 concentration in SLE patients**

**
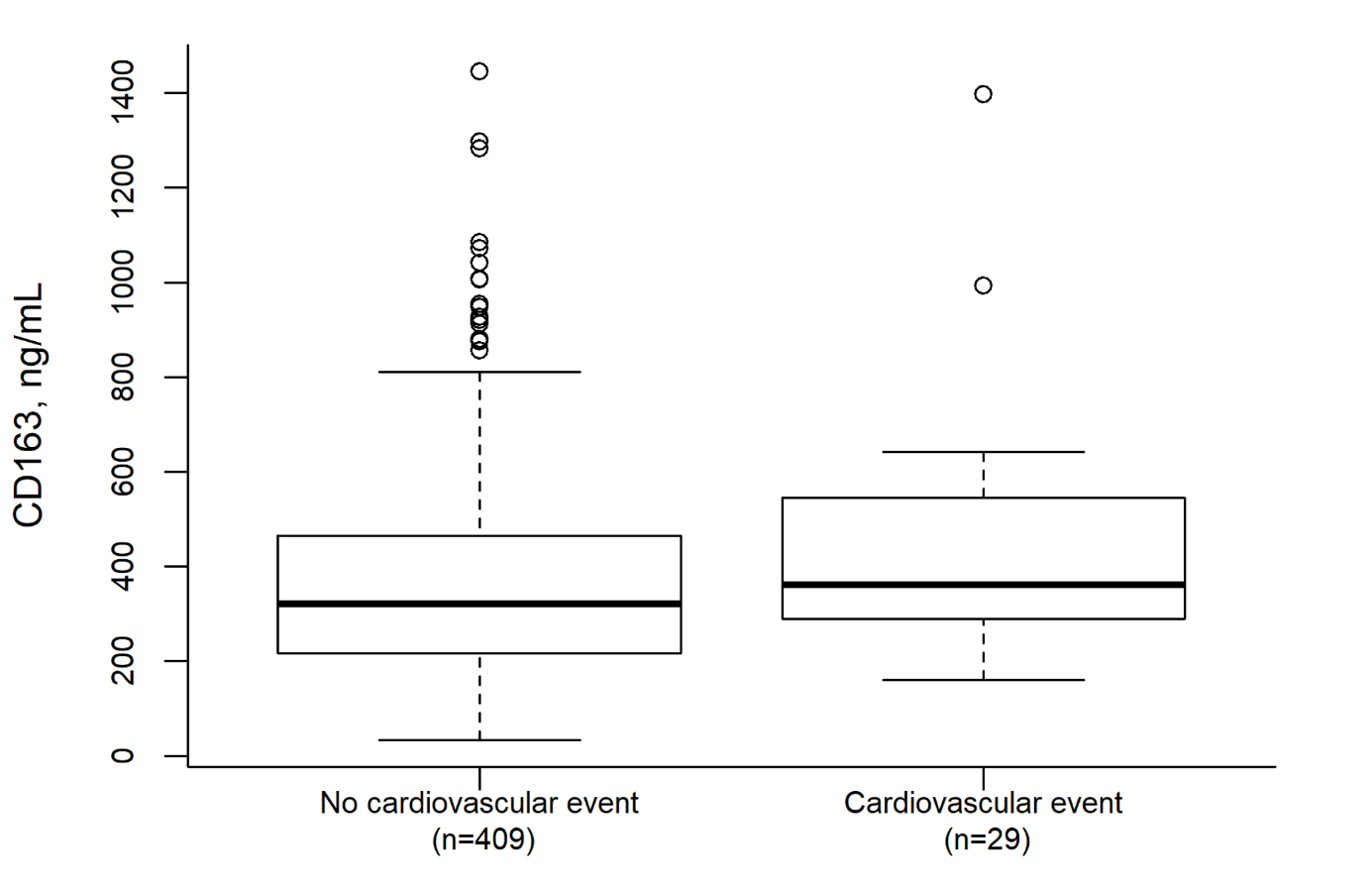
**

Serum level of sCD613 at baseline tended to higher in SLE patients who present CVE during follow-up as compared to those who did not (no CVE) (p=0.0669). Box plots of median level of sCD163 in both groups are represented. Analysis was performed on 438 patients. CVE: cardiovascular event

**Figure S3 Maximally selected Log-Rank statistic for** **the cutpoint in sCD163 value**


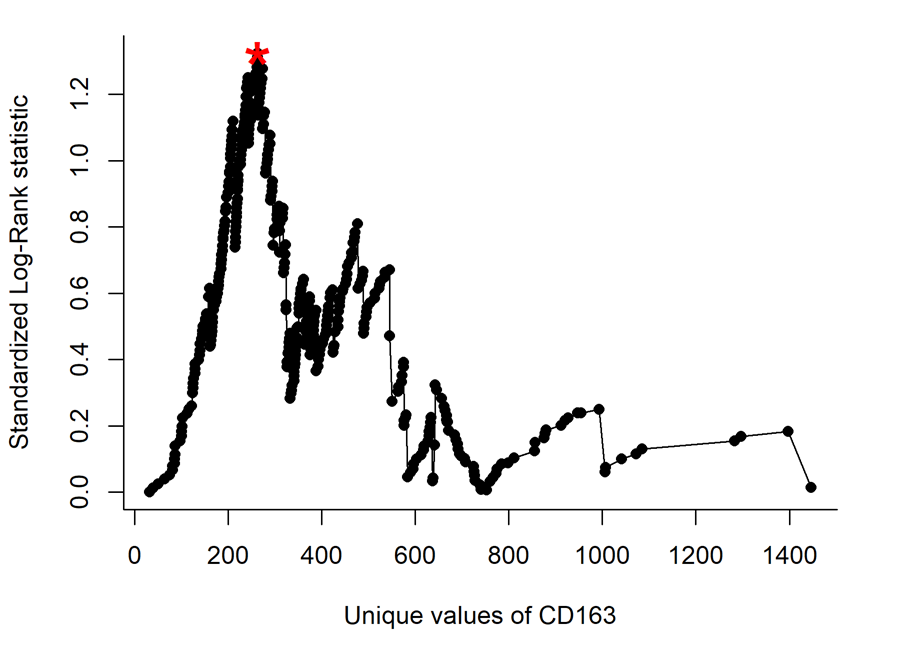


Red dot indicates the best cut-off value to separate patient with and without CVE

**Figure S4** **Figure 2. sCD163 in SLE patients correlates with SLEDAI**

**
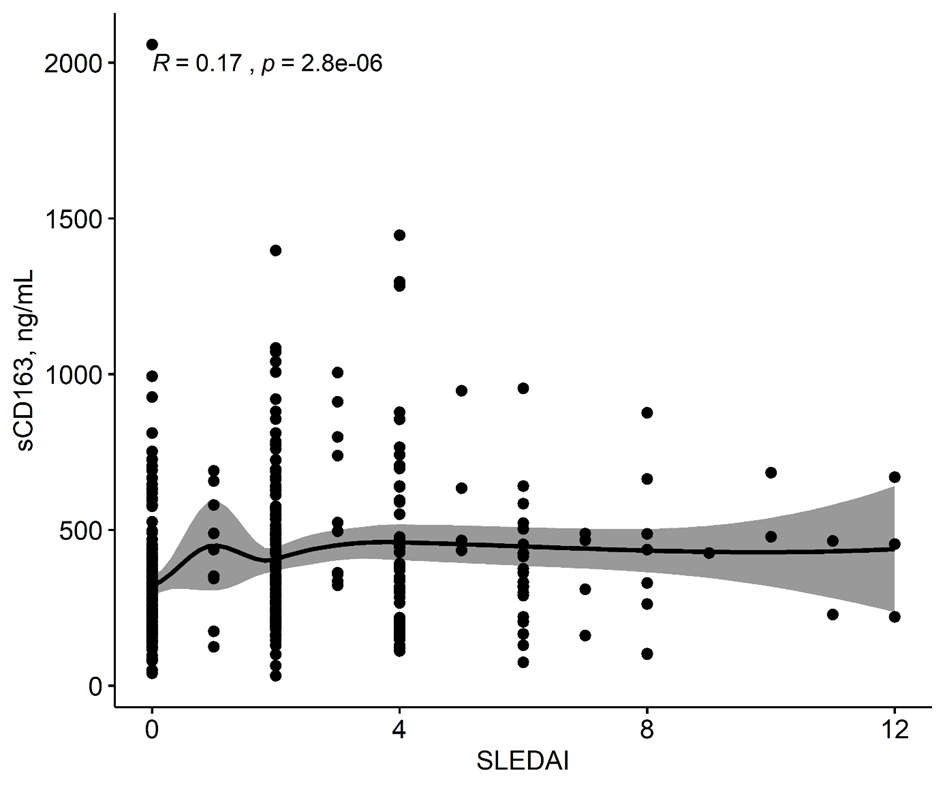
**

The Kendall rank correlation test was used to measure the association between sCD163 measured in serum and SLEDAI activity score both determined at PLUS inclusion

Analysis was performed on 438 patients

SLEDAI: Systemic Lupus Erythematosus Disease Activity Index

**REFERENCE**

1. Costedoat-Chalumeau N, Galicier L, Aumaître O, Francès C, Le Guern V, Lioté F, et al. Hydroxychloroquine in systemic lupus erythematosus: results of a French multicentre controlled trial (PLUS Study). Ann Rheum Dis. 2013;**72**:1786–92
2. Chezel J, Costedoat-Chalumeau N, Laouenan C, Rouzaud D, Chenevier-Gobeaux C, Le Guern V, et al. Highly sensitive serum cardiac troponin T and cardiovascular events in patients with systemic lupus erythematosus (TROPOPLUS study). Rheumatology (Oxford). 2021;**60**:1210-1215.
3. Mach F, Baigent C, Catapano AL, et al. 2019 ESC/EAS Guidelines for the management of dyslipidaemias: lipid modification to reduce cardiovascular risk. Eur Heart J 2020; **41**: 111–88.
4. Miyakis S, Lockshin MD, Atsumi T, et al. International consensus statement on an update of the classification criteria for definite antiphospholipid syndrome (APS). Journal of Thrombosis and Haemostasis 2006; **4**: 295–306.
5. Weening JJ. The Classification of Glomerulonephritis in Systemic Lupus Erythematosus Revisited. Journal of the American Society of Nephrology 2004; **15**: 241–50.
6. Lausen B, Schumacher M. Maximally Selected Rank Statistics. Biometrics 1992; **48**: 73–85.
